# Monotherapy with Metformin versus Sulfonylureas and Risk of Cancer in Type 2 Diabetic Patients: A Systematic Review and Meta-Analysis

**DOI:** 10.1155/2019/7676909

**Published:** 2019-11-19

**Authors:** Abraham Nigussie Mekuria, Yohanes Ayele, Assefa Tola, Kirubel Minsamo Mishore

**Affiliations:** ^1^Department of Pharmacology, College of Health and Medical Sciences, Haramaya University, Harar, Ethiopia; ^2^Department of Clinical Pharmacy, College of Health and Medical Sciences, Haramaya University, Harar, Ethiopia; ^3^Department of Epidemiology and Biostatistics, College of Health and Medical Sciences, Haramaya University, Harar, Ethiopia

## Abstract

**Background:**

Accumulating evidence suggests that patients with type 2 diabetes mellitus and hyperinsulinemia are at an increased risk of developing malignancies. It remains to be fully elucidated whether the use of metformin, an insulin sensitizer, and/or sulfonylureas, insulin secretagogues, affects cancer incidence in subjects with type 2 diabetes mellitus.

**Objective:**

A systematic review and meta-analysis was performed to compare the risk of cancer incidence associated with monotherapy with metformin compared with monotherapy with sulfonylureas in type 2 diabetes mellitus patients.

**Methods:**

Search was performed throughout MEDLINE/PubMed, EMBASE, Google Scholar, Cochrane Central Register of Controlled Trials (CENTRAL), and ClinicalTrials.gov up until December 2018. In this meta-analysis, each raw data (unadjusted) and study-specific (adjusted) relative risks (RRs) was combined and the pooled unadjusted and adjusted RRs with the 95% CI were calculated using the random-effects model with inverse-variance weighting. Heterogeneity among the studies was evaluated using *I*^2^ statistics. Publication bias was evaluated using the funnel plot asymmetry test. The Newcastle-Ottawa scale (NOS) was used to assess the study quality.

**Results:**

A total of 8 cohort studies were included in the meta-analysis. Obvious heterogeneity was noted, and monotherapy with metformin was associated with a lower risk of cancer incidence (unadjusted RR = 0.74, 95% CI: 0.55-0.99, *I*^2^ = 97.89%, *p* < 0.00001; adjusted RR = 0.76, 95% CI: 0.54–1.07, *I*^2^ = 98.12%, *p* < 0.00001) compared with monotherapy with sulfonylurea, using the random-effects model with inverse-variance weighting.

**Conclusions:**

According to this review, the monotherapy with metformin appears to be associated with a lower risk of cancer incidence than monotherapy with sulfonylurea in patients with type 2 diabetes. This analysis is mainly based on cohort studies, and our findings underscore the need for large-scale randomized controlled trials to establish the effect of metformin monotherapy, relative to sulfonylureas monotherapy on cancer.

## 1. Introduction

Type 2 diabetes mellitus (T2DM) is a progressive metabolic disorder that accounts for more than 90% of all cases of DM [1]. It is a chronic metabolic disorder characterized mainly by the presence of insulin resistance, diminished pancreatic beta cell production, and persistently elevated blood glucose levels [2]. The incidence and prevalence of T2DM have been increasing worldwide and currently considered a major health problem. Globally, an estimated 422 million adults were living with diabetes in 2014, compared to 108 million in 1980 [3].

A central aspect of T2DM is the development of chronic micro- and macrovascular complications associated with high morbidity and mortality [4]. Besides, there is a growing evidence base to support a connection between diabetes, predominantly T2DM, and certain types of cancer [5]. Meta-analysis has revealed T2DM to be an independent risk factor for the development of different types of cancer [6].

It is known that most T2DM patients have long-term exposure to multiple antidiabetic drugs. However, frequently prescribed antidiabetic drugs for the treatment of T2DM are metformin and sulfonylurea (SU) derivatives [7]. In light of the increasing prevalence of patients under treatment with these drugs, efforts have been undertaken to elucidate the association between the use of these drugs and cancer risk. However, results from epidemiologic studies have been inconsistent and the reasons underlying this heterogeneity need to be further investigated. Meanwhile, few systematic reviews and meta-analysis are indicating the decreased risk of cancer among T2DM patients taking metformin compared with nonusers. However, currently, there is no published systematic review or meta-analysis that compared the overall risk of cancer associated with the use of metformin monotherapy against sulfonylurea monotherapy in T2DM patients using epidemiological studies. In addition, there are limited published systematic reviews or meta-analysis that compared the site-specific risk of cancer associated with the use of metformin monotherapy versus sulfonylurea monotherapy in T2DM patients using epidemiological studies. Therefore, this review is aimed at appraising published epidemiological studies that involved T2DM patients and reported data on the risk of cancer associated with exposure to metformin monotherapy in comparison with sulfonylureas monotherapy.

## 2. Methods

### 2.1. Study Protocol

Items for systematic review and meta-analysis (PRISMA) flow diagram were followed during the process of article identification to inclusion [8]. A PRISMA checklist was used while conducting the review. The completed checklist is provided as supplementary material ([Supplementary-material supplementary-material-1]).

### 2.2. Data Sources and Search Strategy

Electronic databases were searched including Medline/PubMed, EMBASE, Cochrane Central Register of Controlled Trials (CENTRAL), and ClinicalTrials.gov in December 2018 to locate articles published on the use of metformin and SUs as monotherapy and their association with cancer. The search was limited to original studies conducted on a human subject and reported in English language peer-reviewed journals before December 2019. The search was conducted using MeSH terms and free text for each domain (diabetes mellitus, metformin, sulfonylurea, and cancer). The search terms used were “diabetes mellitus”, “metformin”, “sulfonylurea”, and “cancer”. The MeSH terms and free text words were combined by “AND”, and in each domain, the terms were combined by “OR” and it was tailored for each database. The search terms were tested to check its capacity of locating articles which were consistent with the inclusion criteria. The reference lists of the identified articles were manually scanned to identify any other relevant studies. A Google Scholar search was conducted to identify any other relevant studies including dissertations, reports published from a conference, and prepublication manuscripts.

### 2.3. Screening and Eligibility of Studies

The documents identified from different electronic sources were exported to ENDNOTE reference software version 7.8 (Thomson Reuters, Stamford, CT, USA) with compatible formats. Duplicate documents were removed with the help of ENDNOTE and manually. Each of the documents retrieved was assessed by two authors (AN and YA) independently for eligibility by reading the title and abstract using the preset inclusion and exclusion. This process was followed by retrieval and assessment of the full texts of the relevant citations. The other authors (AT and KM) were involved in resolving the disagreement between two authors who assessed the eligibility of the document.

### 2.4. Inclusion and Exclusion Criteria

We included studies on T2DM patients that reported quantitative data on cancer incidence associated with exposure to monotherapy with metformin, in reference SUs with the monotherapy group. The studies that involve therapies with antidiabetics other than metformin and SUs that reported cancer mortality rate were not included in the review. Articles with no full text (after communicating to corresponding authors) were also excluded.

### 2.5. Data Extraction

Data extraction format prepared in excel was developed to extract data on study characteristics and outcomes. Two authors (YA and AN) independently extracted the data related to study characteristics: study design, country, publication year, sample size, total number of cases, and control in the metformin and SU groups, cancer site, time period, adjustment and stratification variables, event and nonevent, RR (or other association measures), and the corresponding 95% CI.

### 2.6. Quality Assessment of Studies

The quality (internal and external validity) of the study which fulfilled the inclusion criteria was checked before data extraction using critical appraisal tools of the Newcastle-Ottawa scale (NOS) [9]. This scale rates studies based on the following three parameters: selection, comparability, and outcome. Studies can receive a maximum possible score of nine stars and be graded as follows: studies receiving <5 stars are rated as low-quality studies and studies receiving >6 stars are rated as high-quality studies. The quality assessment was performed by two investigators (AN, YA), and any disagreements regarding study quality were resolved by KM.

### 2.7. Outcome Measurements

The main outcome of measures in this meta-analysis was the overall risk of cancer. Site-specific risks of cancer were reported in some of the included studies, but the relatively small number of studies in each type of cancer limited us to conduct secondary analyses.

### 2.8. Data Processing and Statistical Analysis

The data were analyzed using Meta-Essentials 1.0 [10]. The summary RR for exposure to metformin versus SU was the measure of interest. Analyses were performed for overall cancer risks. For analysis of these studies, a random-effects model with inverse-variance weighting was used to pool the raw data (unadjusted) and study-specific (adjusted) RRs and corresponding 95% CIs were reported. Moreover, the total variation across studies that were due to heterogeneity rather than chance were evaluated using the *I*^2^ statistic [11]. Besides, the risk of publication bias was assessed by funnel plots. For all hypothesis tests, evidence was based on *p* < 0.05, and the 95% CIs were therefore presented.

## 3. Results

### 3.1. Search Result

**Figure 1:**
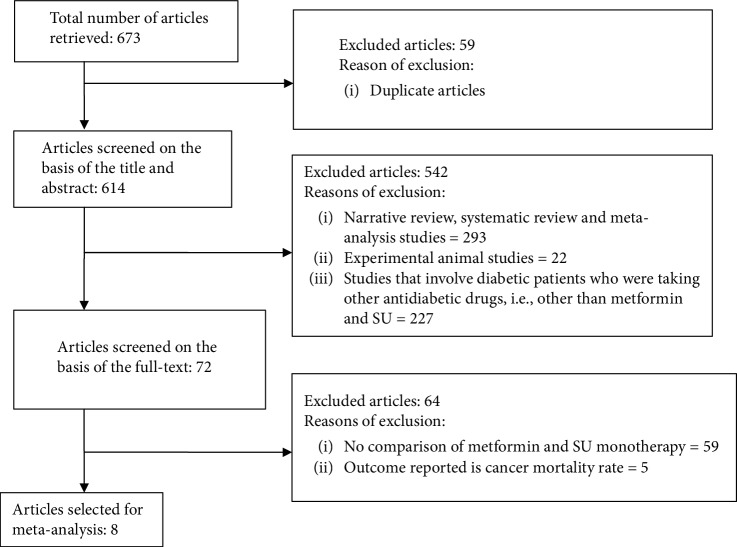
Flow chart of the search result and selection of studies for inclusion in the meta-analysis.

As shown in Figure 1, the initial search yielded 673 articles, of which 59 articles were found to be duplicates. From the 614 remaining papers, 542 were excluded after reading the title and abstract. The remaining 72 articles were considered of interest, and their full text was retrieved for detailed evaluation. Of these, 64 articles were further excluded because they did not satisfy the inclusion criteria. The remaining 8 studies [12–19] complied with the inclusion criteria and were considered for the analysis.

### 3.2. Study Characteristics

All of the eight studies that met the inclusion criteria were cohort studies. As indicated in Table 1, six studies were based on European [12, 13, 15–18], one study was based on USA [19], and the other one study was based on Asian T2DM patients [14]. All the included studies were published between 2009 and 2018. On the other hand, all the included studies have done a comparison of the risk of cancer associated with monotherapy with metformin versus monotherapy with SU in T2DM patients. As shown in Table 1, the sample sizes of the included studies in the metformin and SU groups ranged from 3,963 to 194,357 and 6,072 to 93,415, respectively.

As shown in Table 1, all of the included studies reported raw event data for overall cancer incidence [12–19]. The total number of subjects enrolled in the studies was 760,291 (metformin group = 520,106 (210,861 females and 309,245 males) and SU group = 240,185 (86,551 females and 153,634 males)). The total number of cancer cases was 23,046 (metformin groups = 13,837 (2.7%), SU groups = 9,209 (3.8%)). On the other hand, seven studies reported adjusted estimates of effects and all of the studies adjusted important confounding factors, such as age and sex [12–18].

### 3.3. Study Outcome Measures

**Figure 2:**
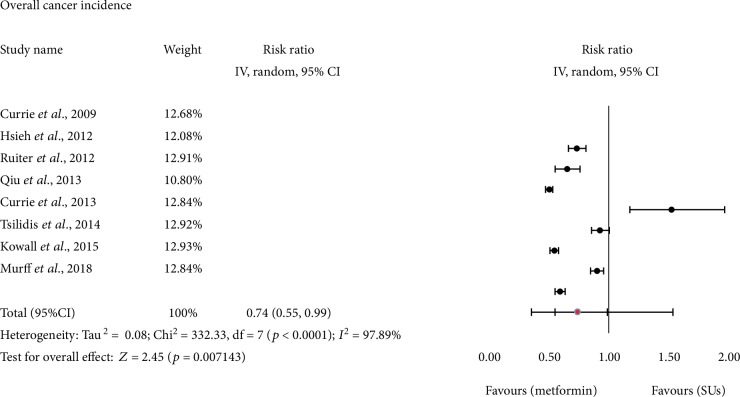
Forest plot of the relative risk of cancer when comparing the use of monotherapy with metformin versus SUs, using the raw data. Black circles represent study-specific relative risk estimates (size of the blue circle reflects the study-specific statistical weight, that is, the inverse of the variance); horizontal lines represent 95% CIs; red circle represents summary relative risk estimates with corresponding 95% CIs; *p* values are from testing for heterogeneity across study-specific raw case data. Abbreviations: CI: confidence interval; SU: sulfonylurea.

After pooling of the eight cohort studies, those that reported raw data for overall cancer incidence suggested significantly lower overall cancer incidence among T2DM patients using metformin monotherapy compared to T2DM patients using SUs as a monotherapy (RR = 0.74, 95% CI: 0.55-0.99, *I*^2^ = 97.89%, *p* < 0.00001; Figure 2).

**Figure 3:**
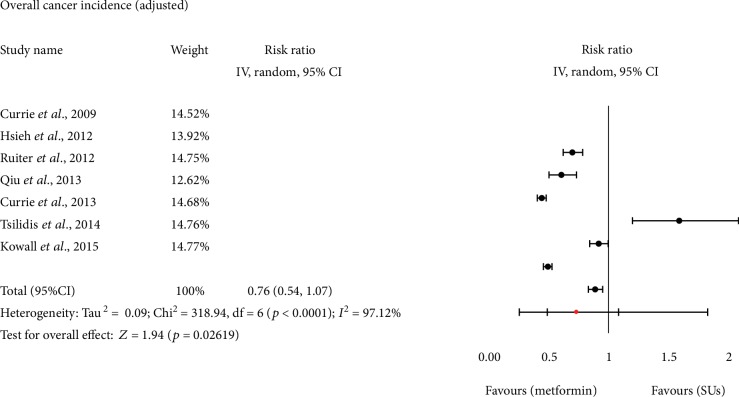
Forest plot of study-specific relative risk estimates (adjusted) overall cancer incidence monotherapy with metformin versus SUs. Black circles represent study-specific relative risk estimates (size of the black circle reflects the study-specific statistical weight, that is, the inverse of the variance); horizontal lines represent 95% CIs; red circle represents summary relative risk estimates with corresponding 95% CIs; *p* values are from testing for heterogeneity across study-specific raw case data. Abbreviations: CI: confidence interval; SU: sulfonylurea.

After pooling of the adjusted estimates, the use of metformin was still associated with a lower risk of cancer incidence than SUs in T2DM patients (RR = 0.76, 95% CI: 0.54–1.07, *I*^2^ = 98.12%, *p* < 0.00001; Figure 3).

**Figure 4:**
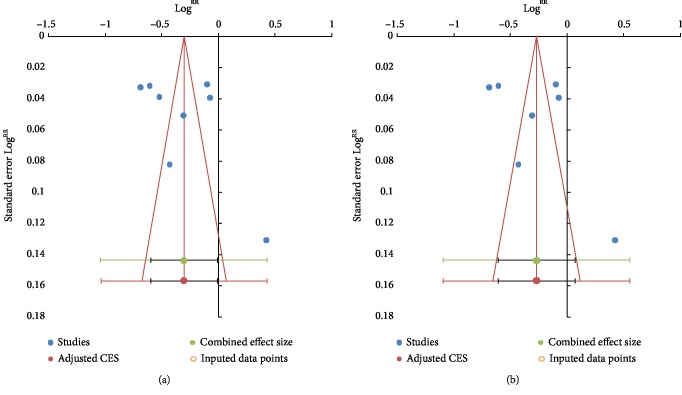
Funnel plot for publication bias in the study investigating the overall risk of cancer associated with the use of metformin monotherapy versus sulfonylurea using raw data (a) and using study-specific (adjusted) RR estimates (b). Abbreviations: RR: relative risk; SE: standard error.

Regarding heterogeneity and publication bias analysis, when pooling the raw data, there was evidence of large heterogeneity (*I*^2^ = 98%, *p* < 0.00001; Figure 4(a)), and evidence for the existence of publication bias (*p* = 0.048). Similarly, in pooling the adjusted RRs, there was still evidence of heterogeneity (*I*^2^ = 98%, *p* < 0.00001; Figure 4(b)), but no evidence for the existence of publication bias (*p* = 0.077).

## 4. Discussion

In this study, the results from the meta-analysis of cohort studies suggested that monotherapy with metformin decreases cancer risk in patients with T2DM compared with SU monotherapy. The current review supports the assumption that metformin potentially has an anticancer effect via the mechanisms suggested by different studies, including impairing cellular metabolism and inhibiting oncogenic signaling pathways, like receptor tyrosine kinase, PI3K/Akt, and mTOR pathways and regulation of cancer stem cells (CSCs) and related pathways as recently reviewed by Saini and Yang [20] and Kheirandish et al. [21]. Moreover, this study is in line with studies that reported a slight increment in cancer risk associated with the use of SUs in T2DM [22, 23]. However, the exact molecular mechanisms connecting SU use to cancer are largely obscure, even though some studies linked them with increased activity of the oncogenic protein insulin-like growth factor- (IGF-) 1 owing to their insulin-secreting effect that has been postulated to promote tumorigenesis both directly and indirectly by acting upon the insulin and IGF-1 receptors expressed on many tumors [24–27].

The major strength of this study is that it was restricted to data with monotherapy of metformin versus SU monotherapy on cancer incidence in patients with T2DM. Moreover, the study pooled the RR using both raw data and study-specific (adjusted) RRs to estimate the overall risk. However, one of the limitations of this study is in that the study was not able to pool the results of other epidemiological studies (randomized controlled trial (RCT) and case-control studies), because of unavailability of enough studies that investigated the risk of cancer in T2DM patients who received metformin monotherapy versus SU monotherapy. Furthermore, few studies have suggested that individual or different generations of SUs may differentially affect cancer risk [28]. However, this study does not examine for the specific effects of individual SU subgroups or different SU generations on cancer risk, since the small sample sizes of those subgroups would have eliminated the study power to detect meaningful associations. On the other hand, the studies have many confounding variables, for instance, age, gender, body mass index, smoking status, alcohol use, comorbidities, and other medication use, and these variables were indeed not uniform in all of the included studies. Hence, it may have made our results less valid. Additionally, cancer risk is related to several lifestyle habits such as smoking, alcohol intake, exercise, and diet [29]; although the studies included adjusted for many of these risk factors, it would have been interesting to analyze the influence of these risk factors on the relationship between the use of antidiabetic medications (metformin and SUs) and cancer risk.

Furthermore, this study is also limited in that the cohort studies included in this analysis may also have time-related biases, particularly immortal-time and time-lag biases. Immortal time is a time during which the outcome under study (cancer) could not have occurred, as metformin or SU-exposed time leads immortal-time related bias. Time-lag bias arises when comparing second- or third-line treatments (SUs or insulin) with first-line (metformin) treatment [30]. In this regard, these patients are unlikely to be at the same stage of the disease. It is, therefore, the results of the studies included in the present analysis that had such kind of bias may mistakenly show that overall cancer risk is lesser or more in the case of metformin or SUs, respectively, as a result of misclassification of the exposure time for these medications. However, this is a major limitation of the observational study design and not the methodology used in the current meta-analysis [22].

## 5. Conclusion

In this review, monotherapy with metformin appears to be associated with a lower risk of cancer incidence than monotherapy with SUs in patients with T2DM. However, further studies with more rigorous study design and data analysis are needed to establish the effect of metformin, relative to SUs on cancer.

## Figures and Tables

**Table 1:** Characteristics of cohort studies included for systematic review and meta-analysis.

First author, year	Country	Study period	Metformin group		Sulfonylurea group		Reported adjusted RR (95% CI)	Adjusting variables
			Case	Total (male, %)	Case	Total (male, %)		
Currie, 2009	UK	2000-2008	1,482	31,421 (51.1)	479	7,439 (54.9)	0.74 (0.65-0.84)	Age, sex, smoking, comorbidity, HbA1c, duration of DM, weight
Hsieh, 2012	Taiwan	2000-2008	199	3,963 (48.3)	470	6,072 (53.8)	0.56 (0.44–0.71)	Age, sex
Ruiter, 2012	Netherlands	1998-2008	1,590	52,698 (46.4)	1,962	32,591 (48.2)	0.90 (0.89-0.91)	Age, sex, number of other drugs used in the year before the start of OGLD, number of hospitalizations in the year before the start of OGLD, calendar time
Qiu, 2013	UK	1995-2008	1,389	39,070 (57.0)	1,165	16,904 (59)	0.93 (0.86-1.02)	Age, sex, duration of T2DM, antidiabetic medication was monotherapy
Currie, 2013	UK	200-2010	2,581	58,532 (55.9)	805	16,218 (54.4)	0.91 (0.83–1.00)	HbA1c, total cholesterol, serum creatinine, BMI, smoking status, antihypertensive-lipid-lowering, antiplatelet therapy, duration of DM, prior history of cancer, LVD, microvascular disease, number of contacts with the GP in the year before the index date, CCI
Tsilidis, 2014	UK	2000-2010	2,303	51,484 (56.1)	1,502	18,264 (57.9)	1.13 (1.05-1.22)	Age, sex, smoking, BMI, alcohol, aspirin, statins, year of 1^st^ antidiabetes prescription, duration of T2DM
Kowall, 2015	Germany & UK	1995-2013	2,965	194,357 (53.8)	1,579	93,415 (56.9)	1.05 (0.99–1.12)	Age at first DM medication, sex, country, time b/n first dx of DM & prescription of first DM drug, obesity, HTN, hyperlipidemia, the prevalence of microcomplications, CCI, use of antihypertensives, antithrombotic agents, aspirin, statins, NSAIDs, & contraceptives
Murff, 2018	USA	2001-2008	1,328	88,581 (95.1)	1,247	49,282 (97.5)	NA	HbA1c, antihypertensive & lipid-lowering medications, & BMI, duration of T2DM

Abbreviation: *N*: number, BMI: body mass index, Cr: creatinine, GP: general practitioner, HbA1c: hemoglobin A1C, CCI: Charlson comorbidity index, NSAIDs: nonsteroidal anti-inflammatory drugs, OGLD: oral glucose-lowering drugs.

## Supplementary Materials

Supplementary Materials PRISMA checklist.doc and Quality assessments of the included studies.doc.
