# Supplementary material for: Monotherapy with Metformin versus Sulfonylureas and Risk of Cancer in Type 2 Diabetic Patients: A Systematic Review and Meta-Analysis
Source: J Diabetes Res. 2019 Nov 19;2019:7676909. doi: 10.1155/2019/7676909 (PMC6885827; doi:10.1155/2019/7676909)
Supplement: Supplementary Materials — PRISMA checklist.doc and Quality assessments of the included studies.doc. [file 7676909.f1.zip › Quality assessments of the included studies.doc]

**Methodological quality of** included studies in the meta-analysis.

| **Quality assessment criteria** | Acceptable (*) | Currie *et al*, 2009 | Hsieh *et al,* 2012 | Ruiter *et al*, 2012 | Currie *et al*, 2013 | Qiu *et al*, 2013 | Tsilidis *et al*, 2014 | Kowall *et al*, 2015 | Murff *et al*, 2018 |
| --- | --- | --- | --- | --- | --- | --- | --- | --- | --- |
| Representativeness of the exposed cohort | Truly & somewhat representative of average adult with T2DM in community | * | * | * | * | * | * | * | * |
| Selection of the comparative cohort | Drawn from same community as SU cohort | * | * | * | * | * | * | * | * |
| Ascertainment of exposure | Secured records, structured interview | * | * | * |  | * | * | * | * |
| Demonstration that outcome of interest was not preset at start of study | Yes |  | * | * | * | * | * | * | * |
| Study controls for age? | Yes | * | * | * | * | * | * | * | * |
| Study controls for at least 3 additional risk factors | BMI, HbA1c levels, ethnicity, family history of cancer, physical activity, smoking, T2DM duration/severity | * |  | * | * |  | * | * | * |
| Assessment of outcome | Independent blind assessment, record linkage | * | * | * | * | * | * |  | * |
| Was follow up long enough for outcomes to occur? | Follow up ≥ 3 years |  | * |  |  | * | * |  | * |
| Adequacy of follow up of cohorts | Complete follow up or subjects lost to follow up unlikely to bias | * |  |  |  |  |  | * |  |
| **Total quality score (0 to 9)** | | 7 | 7 | 7 | 7 | 7 | 8 | 7 | 8 |
